# Flap Valve-Preserving Vertical Sleeve Gastrectomy (INNOVATE-VSG): Clinical Trial Study Protocol

**DOI:** 10.1007/s11695-025-07675-1

**Published:** 2025-02-22

**Authors:** Ninh T. Nguyen, Kishore M. Gadde, Ravinder K. Mittal

**Affiliations:** 1 University of California, Irvine Medical Center, Orange, USA; 2 University of California, San Diego, San Diego, USA

**Keywords:** Gastroesophageal valve, Gastroesophageal reflux disease (GERD), Bariatric surgery, Sleeve gastrectomy, Antireflux barrier

## Abstract

**Background:**

Conventional vertical sleeve gastrectomy (cVSG), the most commonly performed bariatric surgery, is associated with low complications, durable weight loss, and significant improvement of many obesity-related comorbidities. However, numerous studies have reported that patients who underwent the cVSG have worsening or new onset (*de* *novo*) gastroesophageal reflux disease (GERD) which could be related to a negative effect of the operative procedure on the geometry of the gastroesophageal junction impacting on the function of the native gastroesophageal valve. It is imperative to innovate the cVSG procedure because chronic GERD is a debilitating condition associated with increased risk for Barrett’s esophagus and esophageal cancer. INNOVATE-VSG aims to test whether a modified flap valve-preserving VSG (*fvp*VSG), compared to cVSG, will be associated with improvement of preexisting GERD.

**Methods:**

The *fvp*VSG incorporates the following surgical modifications that strengthen the antireflux barrier: achieving 3 cm intrabdominal esophageal length; repair of the diaphragmatic crura; and preservation of 3 cm length of gastric fundus/cardia during the sleeve gastrectomy procedure which will be used to symmetrically wrap around the distal esophagus (120–160° wrap) to restore the naturally occurring gastroesophageal valve. A total of 44 obese patients (BMI 35–50 kg/m^2^) with pathologic GERD, confirmed by abnormal acid exposure time (AET), will be randomly assigned to cVSG or *fvp*VSG in this pilot randomized clinical trial at two academic sites. The primary outcome is the change in AET at 6–9 months after surgery. Secondary outcomes include changes in the lower esophageal sphincter pressure, compliance of the esophagogastric junction, weight loss, and quality of life.

**Discussion:**

Data generated from the INNOVATE-VSG trial will be used to design a larger multi-center randomized clinical trial to confirm the value of preserving a functioning gastroesophageal valve following sleeve gastrectomy.

## Introduction

Bariatric surgery is the most effective treatment for obesity with clinically significant long-term weight loss along with amelioration or resolution of obesity-related comorbidities [1–4]. According to the American Society of Metabolic and Bariatric Surgery (ASMBS), approximately 280,000 operations were performed in the USA in 2022 for bariatric surgery [1]. The majority of these operations were the conventional vertical sleeve gastrectomy (cVSG), accounting for 57.4% of all bariatric operations. The Roux-en-Y gastric bypass (RYGB) is the second most common primary bariatric procedure, accounting for 22.2% of cases [5]. The reasons for the greater use of the cVSG include its procedural simplicity, i.e., no need for a gastrointestinal anastomosis, short operative time, ability to be performed as an outpatient procedure, durable long-term weight loss, and significant improvement of many obesity-related comorbidities. However, many studies have reported an unintended consequence of the cVSG which is worsening or new onset (*de* *novo*) gastroesophageal reflux disease (GERD) following the surgery. Depending upon the method of assessment and the follow-up duration, GERD incidence post-cVSG varied considerably with some studies reporting up to 68% [6–11]. A 2012 International Sleeve Gastrectomy Expert Panel reported an average GERD incidence of 31% after cVSG [9]. A 2020 meta-analysis estimated that 23% of patients developed *de* *novo* GERD after cVSG. [10] DuPree et al. in an analysis of 4832 patients undergoing cVSG found that 84% continued to have GERD symptoms postoperatively [7]. In the SM-BOSS trial conducted in Switzerland, patients with severe obesity were randomly assigned to cVSG (*n* = 107) or RYGB (*n* = 110) [12]. At 5-year follow-up, *de* *novo* GERD was reported by 18/57 (32%) and 6/56 (11%) in the cVSG and RYGB groups, respectively. Among the patients who had preoperative GERD, 32% in the cVSG group had worsening of symptoms compared to 6% in the RYGB group. In the SLEEVEPASS trial conducted in Finland, patients with severe obesity were randomized to either cVSG (*n* = 121) or RYGB (*n* = 119) [13]. GERD symptoms worsened at 10-year follow-up among 44/90 (49%) patients who had cVSG and 8/85 (9%) who had RYGB [13]. Using 24-h impedance, pH study, and manometry testing, Poggi et al. found that among VSG patients, the prevalence of pathological reflux increased from 47.2% preoperatively to 88.7% postoperatively; average DeMeester score increased from 16.7 (normal being < 14.7) to 42.9; LES pressure decreased from 12.3 to 8.9 mmHg, and the stimulated gastric pressure increased from 27.1 to 133.0 mmHg. [14] Although RYGB is an appropriate surgical alternative in the management of patients with obesity and preexisting pathologic reflux, this procedure is associated with a higher risk of complications including the risks for anastomotic marginal ulceration, dumping syndrome, bowel obstruction, and internal herniation, thus reducing its attractiveness as a primary bariatric operation [13, 14].

The GERD after cVSG was thought to be primarily related to the high-pressure, non-compliant system and technical issues associated with narrowing of the gastric incisura leading to a partial, distal gastric obstruction following sleeve gastrectomy [9, 15]. However, despite technical improvements in the construction of the sleeve over the years, GERD continues to plague this surgical procedure. We postulated that GERD development after sleeve gastrectomy is not related to individual skill level of the surgeon but rather due to the anatomic disruption of the antireflux barrier (ARB) as it relates with the specific technique of the cVSG operation, as it currently stands.

**Fig. 1:**
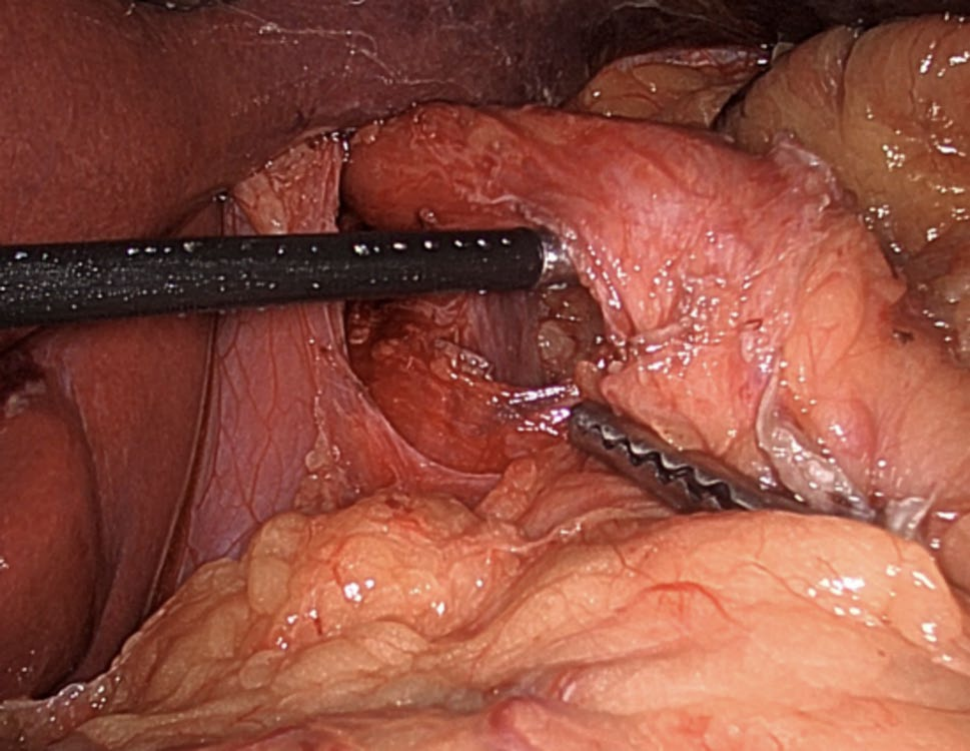
Laparoscopic esophageal hiatus dissection to achieve 3 cm intraabdominal esophageal length and closure of the diaphragmatic crus to strengthen the antireflux barrier

**Fig. 2:**
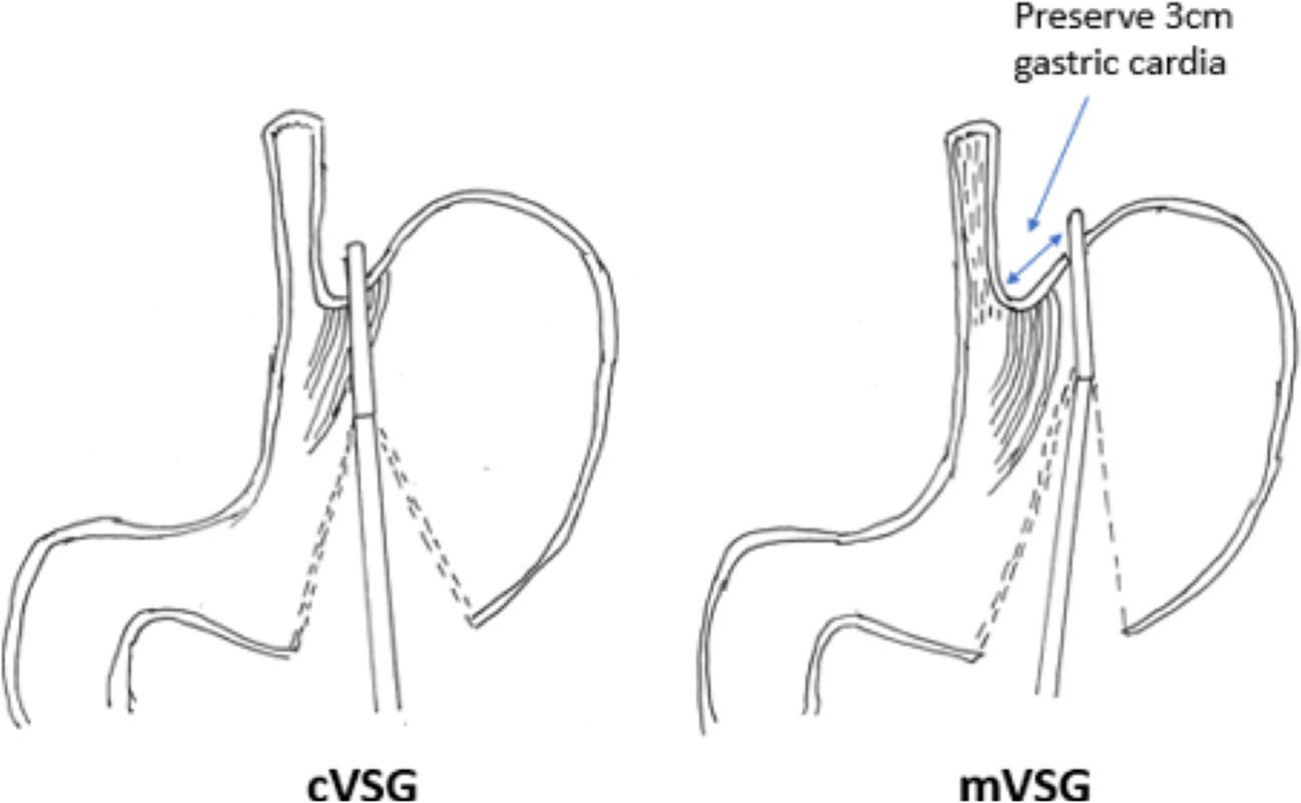
Gastric resection in the cVSG goes directly through the angle of His which disrupts the gastric sling fibers and eliminates the naturally-occurring gastroesophageal valve, defined as an anatomic valve composed of a segment of gastric fundus in direct apposition to the intraabdominal esophagus. In contrast, the *fvp*VSG strengthens the antireflux barrier by including (1) dissection of the hiatus to achieves 3 cm intraabdominal esophageal length, (2) preserving 3 cm of gastric fundus/cardia, and (3) performing partial wrap (120–160°) around the distal esophagus, thus preserving the gastroesophageal valve. The proposed antireflux mechanism of *fvp*VSG is related to the impact of gastric distention acting upon the esophago-gastric high pressure zone

**Fig. 3:**
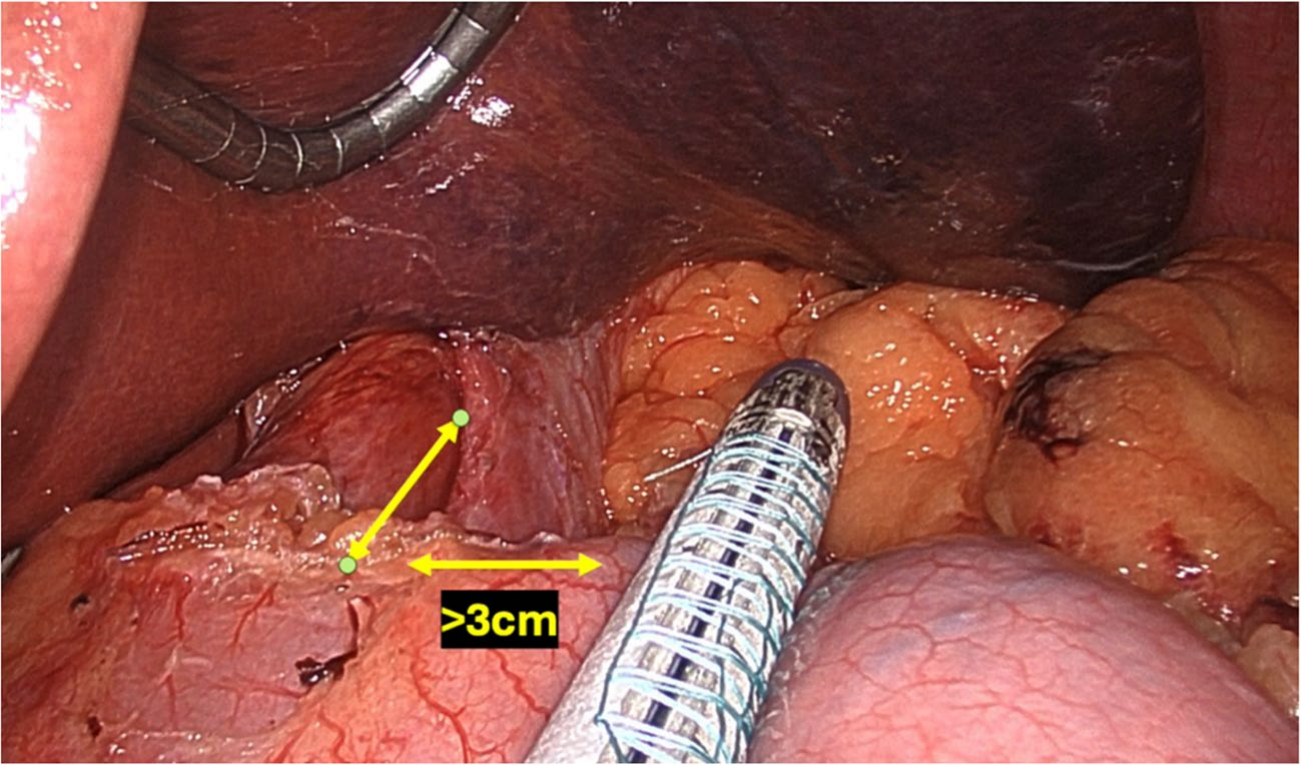
Laparoscopic gastric stapling with preservation of 3 cm gastric fundus/cardia

**Fig. 4:**
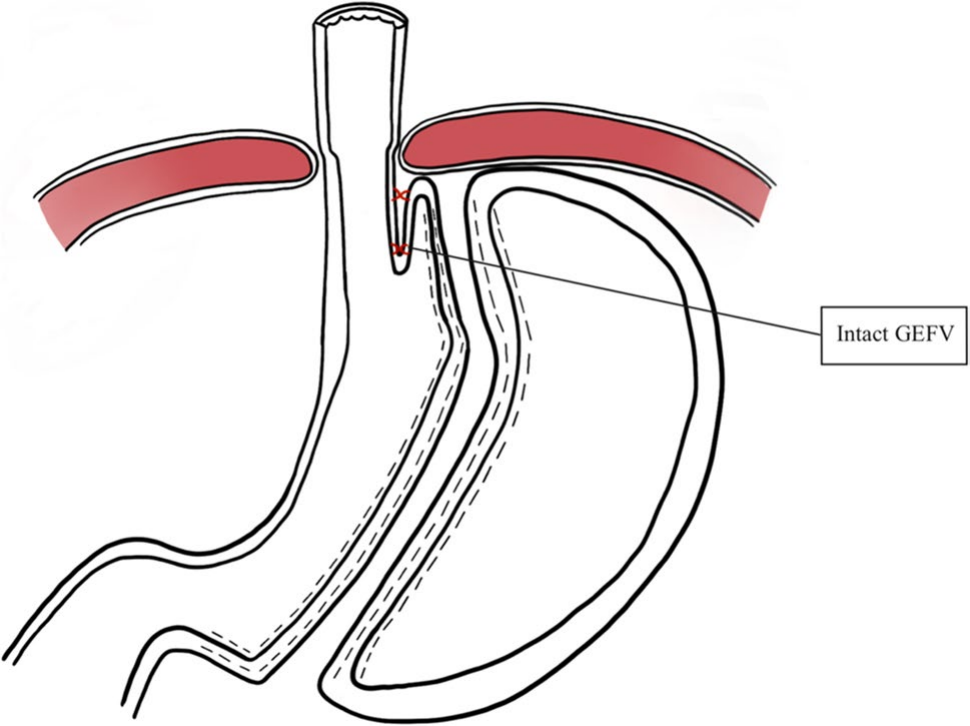
Schematic drawing of the flap valve preserving sleeve gastrectomy

**Fig. 5:**
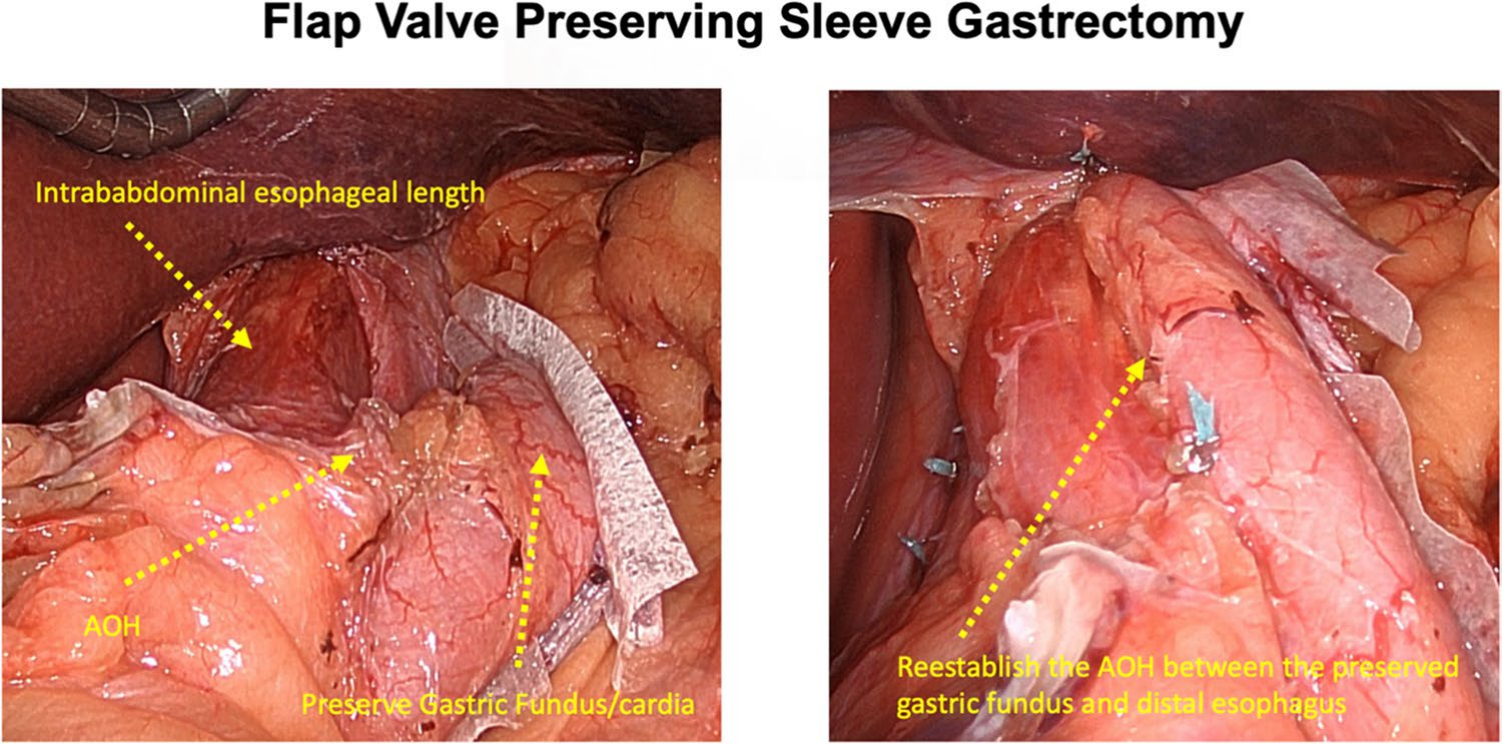
Laparoscopic preservation of the gastric fundus/cardia followed by reestablishing the angle of His and 120–160° partial wrap around the distal esophagus. It should be noted that the wrap occurs between 1-5 o'clock position with a goal to reestablish the naturally-occurring gastroesophageal valve

**Fig. 6:**
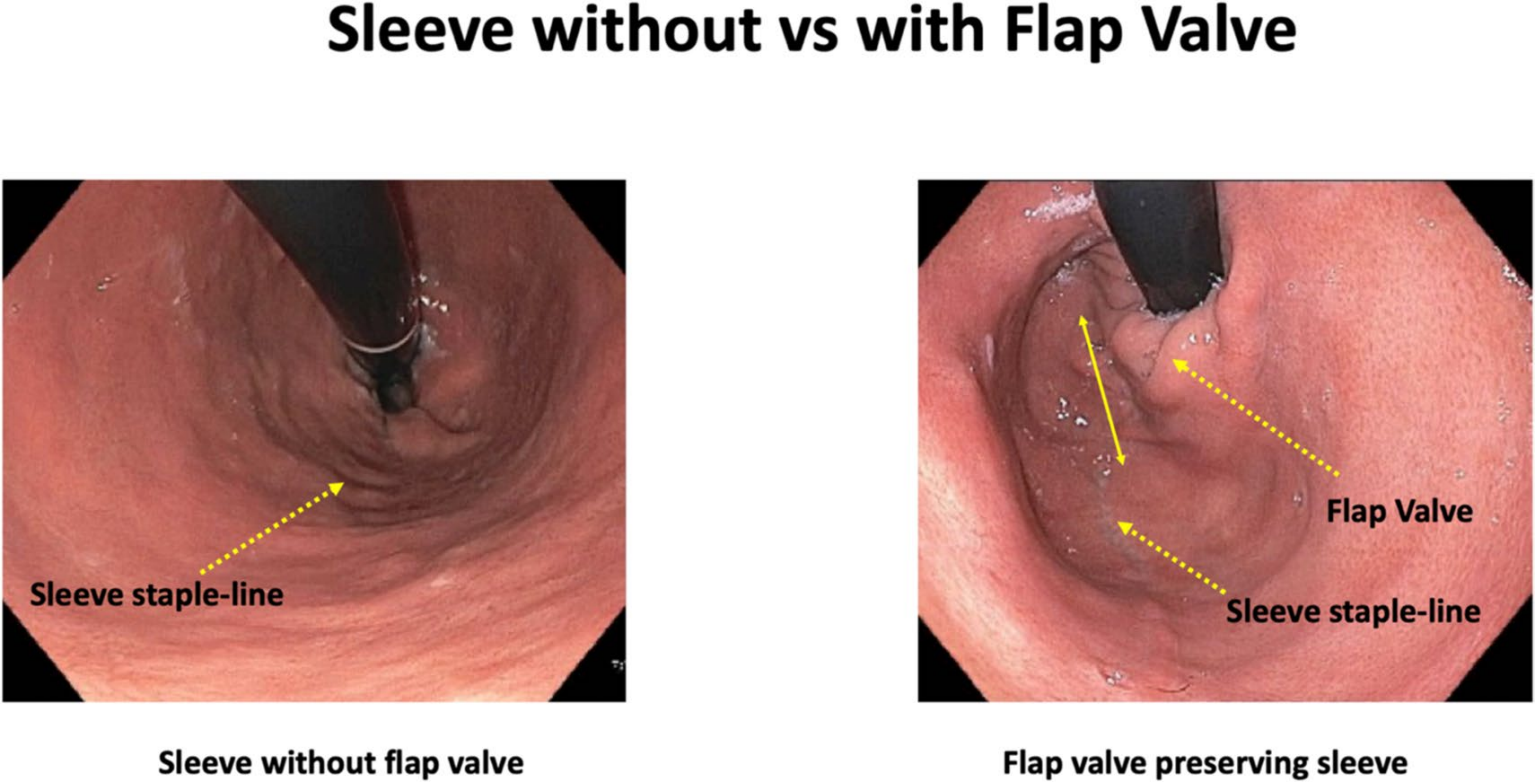
Endoscopic view of cVSG versus *fvp*VSG at 6 months follow-up. Endoscopic view of the *fvp*VSG demonstrates a visible gastroesophageal valve, whereas the cVSG shows a wide hiatal opening without a mechanical antireflux barrier to the esophagus

The cVSG, which reduces the gastric volume, did not consider the importance of avoiding the disruption of the ARB, which can lead to worsening of GERD. One of the native mechanisms protective against reflux is that the esophagus enters on the side of the stomach at an angle which results in the formation of a gastroesophageal valve mechanism. The latter can be seen on the retroflex view of the esophagogastric junction during endoscopic examination. The cVSG resects the entire gastric fundus and essentially creates a continuous tube with the esophagus entering directly into the stomach, thus eliminating the naturally-occurring gastroesophageal valve. Elimination of the gastroesophageal flap valve (GEFV) is one of the most important mechanisms for the increased risk of GERD after cVSG [16]. With the understanding that disruption of the ARB leads to the development of GERD, our trial proposes a surgical modification which is described as a flap valve preserving VSG (*fvp*VSG). The main technical modifications in the proposed *fvp*VSG include surgical principles that strengthen the ARB. Specifically, the technique for *fvp*VSG includes dissection of the esophageal hiatus with increasing the intra-abdominal esophageal length and repair of the diaphragmatic crura, both of which are not commonly performed in the cVSG unless there is a large hiatal hernia (Fig. 1). Additionally, the stapling technique for cVSG is altered, instead of gastric transection line going through the angle of His at the esophagogastric junction; it is performed 3 cm lateral to the angle of His thus preserving a small portion of the fundus/cardia (Figs. 2 and 3). This modification prevents disruption of the gastric sling fibers as shown in Fig. 3. Additionally, the preserved gastric fundus/cardia allows it to be wrapped around the distal esophagus (120–160° fundoplication) between 1 and 5 o' clock position, which we believe recreates the angle of His and preserves the GEFV (Figs. 4 and 5). This newly constructed gastroesophageal complex preserves the gastroesophageal valve that can act as a barrier to reflux. The latter can be visibly seen on endoscopic exam at follow-up (Fig. 6). The technical modifications described above align with the well-known principles of antireflux surgery (i.e., Nissen fundoplication) in the management of pathologic reflux. However, this technical innovation is not considered as a conventional fundoplication procedure combined with a sleeve gastrectomy as the preserved gastric fundus/cardia is too small to effectively perform any standard fundoplication procedures (i.e. Nissen, Rossetti-Nissen, Toupet, Dor, ect.). The goal for this innovation is more to reestablish the naturally-occurring flap valve that acts as an excellent antireflux barrier [16]. The proposed antireflux mechanism of the *fvp*VSG is related to the impact of gastric distention acting upon the esophageal high pressure zone [17]. In summary, the proposed *fvp*VSG is a technical innovation that incorporates the principles of antireflux surgery into cVSG to minimize GERD.

## Methods and Study Research Design

INNOVATE-VSG is a randomized, single-blind, parallel-design clinical trial with a 12-month follow-up, anticipated to enroll patients at two sites. A total of 44 subjects with BMI of 35–50 kg/m^2^, meeting eligibility for bariatric surgery, and having concomitant pathologic GERD (defined as acid exposure time [AET] of ≥ 4.9% as assessed by the Bravo pH study) will be assigned in 1:1 ratio to cVSG or *fvp*VSG. Recruitment is competitive, i.e., one site may recruit more patients than the other. Only the surgeons and the statistician will be unblinded to the randomized treatment assignment; others including the staff performing mechanistic assessments will remain blinded throughout the trial. As is the standard practice at both sites, the patients will have pre- and post-surgery assessments and non-surgical ancillary interventions including diet and lifestyle counseling.

The primary aim is to determine whether *fvp*VSG will be associated with lower acid exposure to the esophagus as assessed by the change in the total AET% at month 6–9 relative to AET% at baseline, as assessed by the Bravo pH study.

To elucidate the mechanistic basis for the primary aim, the following tests will be performed pre-operatively and 6–9 months post-surgery: (1) high-resolution esophageal manometry (HREM) [18] to assess the LES pressure and intragastric pressure; (2) impedance planimetry (EndoFLIP) [19] to assess changes in the gastroesophageal junction compliance; and (3) assess the length of the flap valve via the retroflex view on endoscopic examination at follow-up [18, 19]. Additionally, the resected stomach specimen will be examined for the presence of gastric sling fibers. The impact of change in GERD symptoms on quality of life (QoL) will be examined with two validated rating scales—GERD-HRQL and SF-36 [20, 21]. Body weight and serum metabolic panel (standard testing for bariatric surgery patients) will also be assessed pre-operatively and at 12-month follow-up. All inclusion and exclusion criteria are listed on Table 1.

**Table 1:** Study inclusion and exclusion criteria

Inclusion criteria	Exclusion criteria
1. Male and female subjects aged 18–60 years 2. BMI 35–50 kg/m^2^ 3. Must meet the BMI criteria before and after 6 months of nonsurgical weight management 4. Presence of abnormal acid exposure time (AET) of 4.9% or above as assessed with the Bravo pH test 5. Have health insurance which pays for the costs of bariatric surgery and standard medical care before and after surgery 6. Women of childbearing potential must be using appropriate contraception to avoid pregnancy throughout the study 7. Must be able to provide written informed consent	1. Hiatal hernia > 2 cm 2. Evidence of significant major esophageal motility disorder 3. Severe gastroparesis 4. Previous bariatric or anti-reflux procedure 5. Barrett’s esophagus 6. Subjects requiring mesh treatment at time of procedure 7. Severe heart/lung disease (e.g., heart failure, unstable coronary artery disease, end-stage lung disease) 8. Subjects with pacemakers, implantable defibrillators, neurostimulators 9. Portal hypertension or cirrhosis 10. Chronic pancreatitis 11. Active cancer treatment 12. Inability to tolerate general anesthesia 13. Uncontrollable coagulopathy 14. Significant and uncontrolled inflammatory bowel disease 15. Severe and/or uncontrolled psychiatric disorder 16. Suicidal ideation or unstable/untreated major depressive disorder within the past year 17. Alcohol or substance use disorder within the past year 18. Pregnant, breastfeeding or plan pregnancy in the coming 24 months 19. Diminished intellectual capacity to consent or follow pre- and post-surgery instructions 20. History of, or any current health condition that would make the subject ineligible for sleeve gastrectomy, or put the subject at risk by participation in the study

### Study Objectives

*Primary endpoint*

- Change in the total AET% at 6–9 months, relative to pre-surgery AET%, as assessed with the Bravo pH test.

*Secondary endpoints:*

- Changes in LES pressure, assessed by HREM
- Changes in gastroesophageal junction compliance, assessed by EndoFLIP
- Assess the length of the flap valve via the retroflex view on endoscopic examination
- Changes in health-related quality of life as assessed with GERD-HRQL and the SF-36 questionnaires
- Changes in GERD symptoms assessed with the GerdQ questionnaire
- Percent total bodyweight loss at month 12
- Proton pump inhibitor (PPI) use

*Safety study endpoints:*

- Adverse and serious adverse events
- 30-day and 12-month complications

### Statistical Analysis

Primary and secondary analyses will be performed using an intent-to-treat (ITT) protocol (i.e., all randomized subjects). Additional sensitivity analyses using modified ITT protocols will be implemented with adjustment for missing data. Per-Protocol analysis will also be conducted to test intervention effect under optimal conditions. The primary efficacy outcome (also used for power analysis) is the change in AET% at month 6–9 relative to AET% at baseline which will be analyzed using an analysis of covariance (ANCOVA) model with fixed effects for treatment group, age, sex, race, baseline weight, and baseline AET%. Secondary analyses will include (1) HREM measures and (2) EndoFLIP measures. Additional analyses will include, but are not limited to, GerdQ score, proportions of subjects with GERD improvement or remission, proportions of subjects with *de* *novo* GERD, proportions of subjects with worsening of GERD based on symptom score, proportions of subjects with shifts in AET% categories, DeMeester score, quality of life measures (GERD-HRQL, SF-36), and percent weight loss. Dichotomous outcomes will be analyzed using a generalized linear model (GLM), specifically, a log-binomial regression model with fixed effects for treatment, strata, and covariates described above. For each between-treatment group comparison of interest, the estimated risk ratio (relative risk), standard error, 95% Wald confidence interval, and *p*-value will be presented. Logistic regression models will also be explored to generate odds ratios to allow for comparability to existing literature regarding efficacy of pharmacologic interventions. The weight loss measure as a continuous variable will be analyzed using linear mixed models (LMM). All secondary and exploratory outcomes will be analyzed using similar models described above (ANCOVA, GLM, and LMM) under the ITT, modified ITT, and per-protocol procedures.

## Discussion

Sleeve gastrectomy is the most frequently performed bariatric operation in the world. This operation is associated with significant weight loss and improvement/resolution of comorbidities. However, there is one side effect that continues to plague this operation, i.e., persistent or de novo GERD. The GERD symptoms can worsen after sleeve gastrectomy and a proportion of patients develop new onset GERD symptoms following surgery. Considering our knowledge of the antireflux barrier, we propose that GERD after sleeve gastrectomy is due to the anatomic disruption of the native antireflux barrier. Specifically, the cVSG transect the gastric fundus at the level of the angle of His, which disrupts the native oblique entry of the esophagus to a direct entry path into the stomach, thus eliminating the naturally-occurring gastroesophageal valve. The proposed *fvp*VSG is a technical variant of the cVSG; it maintains the weight loss function of a bariatric operation while strengthening the antireflux barrier at the same time. Our findings could have an impact of reducing the prevalence of GERD following VSG.

## Trial Status

This trial is anticipated to open in February 2025.

## Data Availability

No datasets were generated or analysed during the current study.
